# Umgang mit psychischer Belastung bei Gesundheitsfachkräften im Rahmen der Covid-19-Pandemie

**DOI:** 10.1007/s00115-020-00905-0

**Published:** 2020-03-27

**Authors:** Moritz Bruno Petzold, Jens Plag, Andreas Ströhle

**Affiliations:** Klinik für Psychiatrie und Psychotherapie, Campus Charité Mitte, Charité – Universitätsmedizin Berlin, corporate member of Freie Universität Berlin, Humboldt-Universität zu Berlin, and Berlin Institute of Health, Charitéplatz 1, 10117 Berlin, Deutschland

**Keywords:** Corona, Ärzte, Pflege, Psychische Gesundheit, Stress, Psychische Belastung, Corona, Physicians, Nurses, Mental Health, Stress, Psychological Distress

## Abstract

**Hintergrund:**

Im Rahmen der aktuellen Covid-19-Pandemie stehen Gesundheitsfachkräfte aller Berufsgruppen vor großen Herausforderungen in der Bewältigung der Krise. Dabei treten zahlreiche außergewöhnliche Stressoren und Risiken nicht nur für die körperliche, sondern auch die psychische Gesundheit der Gesundheitsfachkräfte auf.

**Ziel der Arbeit:**

Der Artikel fasst erste Empfehlungen zur Reduktion von Stress und psychischer Belastung bei Gesundheitsfachkräften im Rahmen der aktuellen Covid-19-Pandemie zusammen.

**Methode:**

Dargestellt werden Empfehlungen zur Reduktion von Stress und psychischen Belastungen bei Gesundheitsfachkräften und wichtige Aspekte, die Führungskräfte zur Reduktion von Stress und psychischer Belastung bei ihren Mitarbeiter*innen beachten sollten. Insbesondere werden die Empfehlungen der Weltgesundheitsorganisation, des Internationalen Roten Kreuzes und der Vereinten Nationen berücksichtigt.

**Ergebnisse:**

Eine Normalisierung psychischer Belastungen, eine ausreichende Befriedigung der Grundbedürfnisse, soziale Unterstützung, eine klare Kommunikation und Aufgabenverteilung und flexible Möglichkeiten zur Arbeitsgestaltung und Inanspruchnahme von Hilfsangeboten ohne Stigmatisierung scheinen besonders wichtige Maßnahmen zu sein.

**Diskussion:**

Der Artikel verschafft Gesundheitsfachkräften und ihren Führungskräften einen ersten Überblick über wichtige Faktoren zum Erhalt der psychischen Gesundheit während der Covid-19-Pandemie.

## Abstract (en)

**Background:**

In the current COVID-19 pandemia, health workers from all professions are facing great challenges in coping with the crisis. In this situation health professionals are confronted with specific stressors and risks not only for the physical but also for the mental health.

**Objective:**

This article summarizes the first recommendations for the reduction of stress and psychological burden in health professionals during the current COVID-19 pandemia.

**Methods:**

The recommendations of the World Health Organization, the United Nations and the International Red Cross Society are summarized. Recommendations for the reduction of stress and psychological distress in health professionals as well as important factors that team leaders can consider in order to reduce psychological distress in employees are presented.

**Results:**

The normalization of strong emotions and stress, the fulfillment of basic needs, social support, clear communication and distribution of tasks, flexible working hours and the utilization of psychosocial and psychological help without stigmatization seem to be particularly important measures.

**Conclusion:**

This article presents guidelines for health professionals and team leaders in healthcare that help to maintain mental health during the COVID-19 pandemia.

## Hintergrund

Der ursprünglich zuerst im Dezember 2019 in der Region Wuhan in China aufgetretene neuartige Virus Covid-19 hat sich seitdem mit großer Geschwindigkeit international verbreitet und wird von der Weltgesundheitsorganisation (WHO) inzwischen als Pandemie eingestuft [3]. Dabei hat sich das Virus inzwischen über 100 Länder verbreitet und die WHO stuft die Pandemie als großes Risiko für die internationale Gesundheit ein [8]. Die Pandemie stellt dabei Gesundheitssysteme weltweit vor große Herausforderungen. Gesundheitsfachkräfte aller Berufsgruppen sind in der Bewältigung der Pandemie stark gefordert. Dabei treten für diese zahlreiche psychische Belastungen und Stressfaktoren auf, die potenziell die psychische Gesundheit der Gesundheitsfachkräfte beeinträchtigen könnten [1]. So treten neben allgemeinen Stressoren auch pandemiespezifische Stressoren auf [4] wie beispielsweise:

- das Risiko, sich und andere zu infizieren, insbesondere in einer Situation, in der die Übertragung des Virus noch nicht vollständig geklärt ist,
- die Fehlinterpretation von Symptomen anderer Erkrankungen (z. B. einer Erkältung) als Symptome einer Covid-19-Erkrankung mit resultierenden Ängsten, infiziert zu sein,
- die Sorge um Familienangehörige und Kinder, die zuhause allein sind, z. B. infolge von Schulschließungen,
- die Sorge vor Verschlechterungen der physischen und psychischen Gesundheit bei Gesundheitsfachkräften, die vorbestehende Erkrankungen oder Risikofaktoren aufweisen.

Dabei stellt das Auftreten von Stress und psychischen Belastungen und den Bedingungen der aktuellen Pandemie eine weitgehend normale Reaktion auf ein außergewöhnliches Ereignis dar. Im Zusammenhang mit der aktuellen Covid-19-Pandemie ist daher bei Gesundheitsfachkräften, wie bei anderen Bevölkerungsgruppen, ebenfalls mit dem Auftreten von unter anderem folgenden Reaktionen zu rechnen [4]:

- Angst, zu erkranken oder zu versterben,
- Angst vor sozialer Isolation, wenn man mit der Erkrankung in Verbindung gebracht wird,
- Gefühle von Hilflosigkeit, Bezugspersonen nicht beschützen zu können und Sorgen, dass Bezugspersonen versterben könnten,
- Angst vor Trennung von Bezugspersonen infolge von Isolations- oder Quarantänemaßnahmen,
- Gefühle von Hilflosigkeit, Langeweile und depressive Symptome infolge von Isolation oder Quarantäne,
- Reaktivierung bedrohlicher Erfahrungen aus früheren Epidemien oder Gesundheitskrisen größeren Ausmaßes.

Zusätzlich sind Gesundheitsfachkräfte spezifischen weiteren Stressoren während der Covid-19-Epidemie ausgesetzt [2, 4, 6]:

- das Erfahren von Stigmatisierung, die Menschen, die mit an Covid-19 erkrankten Patient*innen arbeiten, entgegengebracht wird (z. B. aufgrund von Sorgen Anderer, die Gesundheitsfachkräfte könnten selbst infiziert sein),
- strikte Sicherheitsmaßnahmen wie das Tragen von Schutzkleidung, die dauerhafte Notwendigkeit von Konzentration und Wachsamkeit sowie stark regulierte Verfahrensanweisungen, die Spontanität und Autonomie einschränken, und die Reduktion körperlicher Berührungen,
- höhere berufliche Belastungen (längere Arbeitszeiten, mehr Patient*innen, hoher Weiterbildungsdruck),
- reduzierte soziale Unterstützung infolge langer Arbeitszeiten und Stigmatisierung von Gesundheitsfachkräften im Umgang mit Covid-19-Patient*innen,
- reduzierte Selbstfürsorge infolge Zeit- und Energiemangel,
- unzureichende Informationen über die Konsequenzen einer längerfristigen Exposition gegenüber Covid-19-infizierter Patient*innen,
- Sorgen, die eigene Familie und Bezugspersonen mit Covid-19 anstecken zu können,
- Konfrontation mit Ärger und Wut gegen die Regierung oder das Gesundheitssystem durch Patient*innen,
- Gefühle von Isolation durch die Separation von dem Team, mit dem man üblicherweise arbeitet,
- Sorgen, dass die Kolleg*innen mit zusätzlicher Arbeit konfrontiert werden, falls man sich selbst in Quarantäne befindet.

Wenngleich das Auftreten von Stress, psychischer Belastung und negativen Emotionen unter den aktuellen Umständen grundsätzlich als normale Reaktion zu bewerten ist, haben die Belastungen, denen Gesundheitsfachkräfte im Rahmen ihrer Arbeit in Zeiten der Covid-19-Pandemie ausgesetzt sind, grundsätzlich das Potenzial, das Auftreten psychischer Erkrankungen wie beispielsweise Angststörungen, Depression oder Traumafolgestörungen zu fördern [1]. So wurden beispielsweise im Rahmen des letzten SARS-Ausbruchs in China erhöhte Level von Stress, Ängsten, Depression und allgemein psychischer Belastung bei Gesundheitsfachkräften festgestellt [11, 12].

Im Rahmen früherer Epidemien, beispielsweise des Ebola-Ausbruchs von 2013 bis 2016 in Westafrika, zeigte sich, dass psychische Belastungen und Ängste in einer solchen Situation eine zentrale Rolle spielen können und nicht nur die Verbreitung einer Erkrankung fördern, sondern auch zu Einschränkungen im Gesundheitssystem führen können, wenn Gesundheitsfachkräfte infolge dieser Belastungen nicht mehr zur Arbeit erscheinen [7].

## Methode

Vor dem Hintergrund der zahlreichen potenziellen Belastungen haben internationale Organisationen erste Empfehlungen zur Reduktion der psychischen Belastung bei Gesundheitsfachkräften während der Covid-19-Pandemie veröffentlicht. Wenngleich umfassendere Handlungsempfehlungen und eine systematische Erforschung der psychischen Belastung von Gesundheitsfachkräften im Rahmen der Covid-19-Pandemie dringend notwendig erscheinen, stellen diese doch eine Möglichkeit zur ersten Orientierung an Maßnahmen, die sich in bisherigen Krisen bewährt haben, dar.

Der vorliegende Artikel fasst insbesondere die Empfehlungen der WHO [10] und des Inter-Agency Standing Committee (IASC) der Vereinten Nationen [4] zusammen.

## Hinweise zum Umgang mit Stress und psychischen Belastungen bei Gesundheitsfachkräften

**Figure 1:**
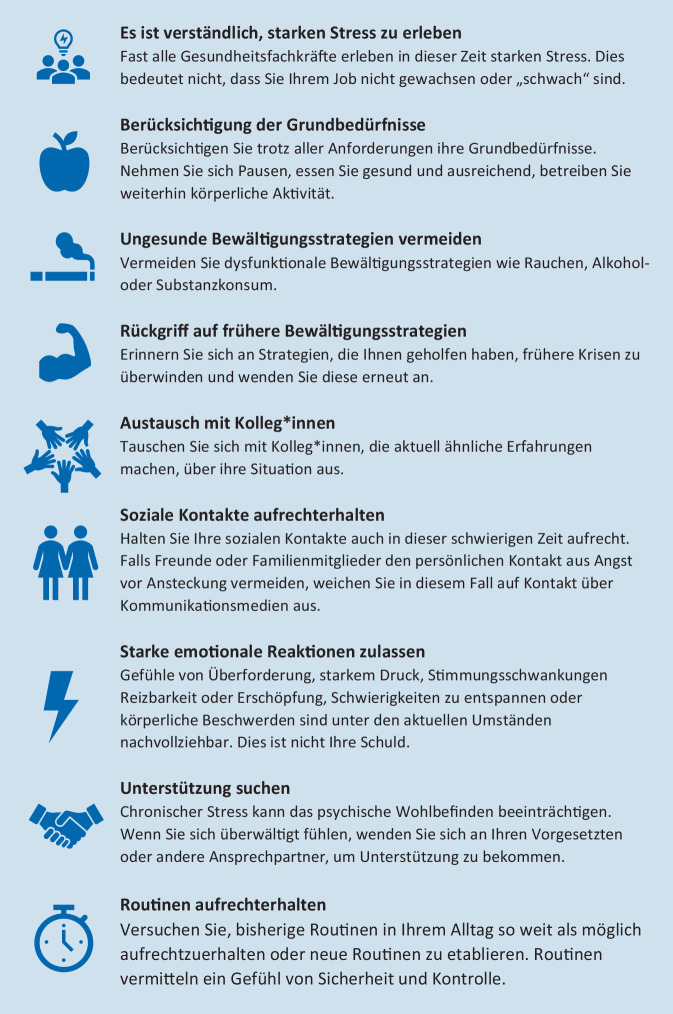

In Abb. 1 sind die aktuellen Empfehlungen der Weltgesundheitsorganisation [10] und des IASC [4] sowie Aspekte der psychologischen Ersten Hilfe des Internationalen Roten Kreuzes [6] zusammengefasst.

Dabei ist es zunächst wichtig, sich bewusst zu machen, dass unter den aktuellen Umständen das Erleben von Stress, Überforderung und heftigen Emotionen eine normale Reaktion darstellt und nicht bedeutet, dass man seinem Job oder den Anforderungen nicht gewachsen oder „schwach“ ist [10]. Dabei sollten heftige Emotionen, auch von Ärger, Reizbarkeit oder Stimmungsschwankungen, als nachvollziehbar betrachtet und nicht schuldhaft verarbeitet werden. Auch unter den aktuell extremen Bedingungen ist es dabei wichtig, auf eine Erfüllung der basalen Grundbedürfnisse zu achten, sich ausreichend Pausen zu nehmen, auf eine gesunde Ernährung zu achten und weiterhin körperlich aktiv zu sein [10]. Zudem sollten zur Bewältigung der Belastung Bewältigungsstrategien, die in früheren Krisen als hilfreich erlebt wurden, reaktiviert werden, dagegen sollte auf einen Rückgriff auf Substanzkonsum als Bewältigungsstrategie verzichtet werden [4]. Zudem kann ein Austausch mit Kolleg*innen, die ähnliche Belastungen erleben und bei denen ein freier Austausch meist auch ohne Einschränkungen durch den Datenschutz möglich ist, helfen, die psychische Belastung zu reduzieren [4]. Das Aufrechterhalten sozialer Kontakte auch im privaten Bereich stellt eine äußerst wichtige Komponente für den Erhalt der psychischen Gesundheit dar. In Fällen, in denen dies aufgrund von Isolations‑/Quarantänemaßnahmen oder Ängsten vor einer Ansteckung nicht möglich ist, sollte auf Telekommunikation, z. B. per Telefon oder Messenger-Dienste, zurückgegriffen werden [4]. Um Gefühle von Kontrolle und Sicherheit zu erlangen, sollten bestehende Routinen aufrechterhalten oder neue Routinen etabliert werden [6].

Darüber hinaus kann auch die Erinnerung daran, zu einem Team zu gehören und eine bedeutungsvolle Aufgabe zu erledigen, zu einer besseren Bewältigung der psychischen Belastung beitragen [6].

## Hinweise für Führungskräfte zur Unterstützung ihrer Mitarbeiter

**Figure 2:**
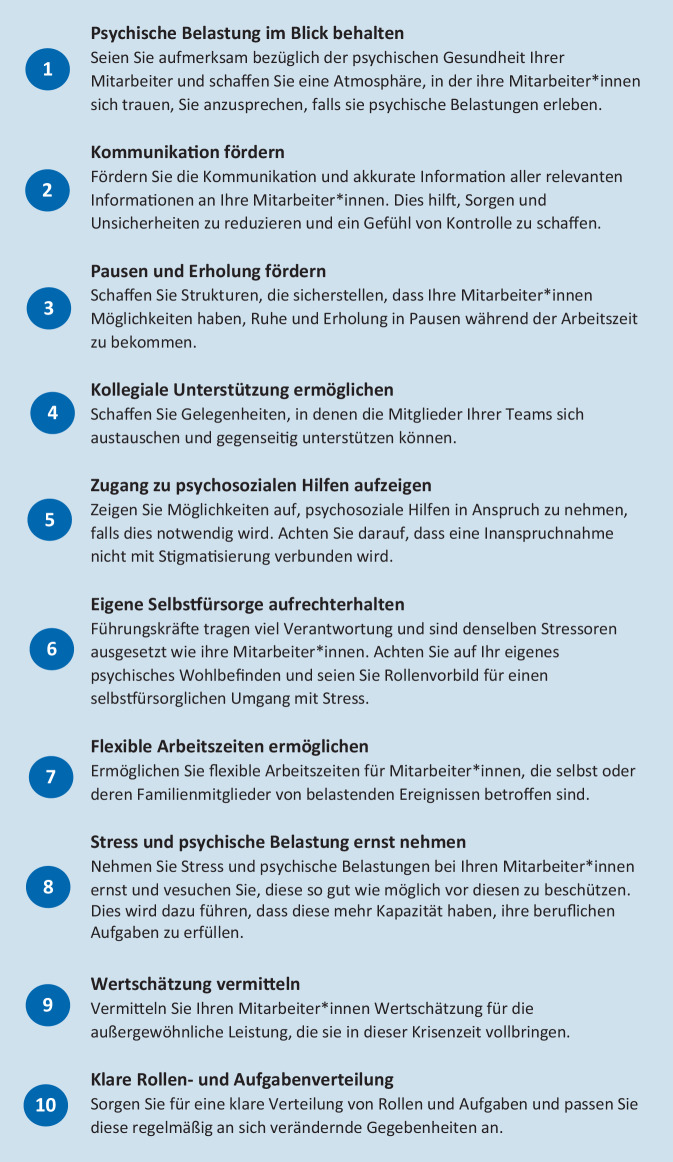

Die Hinweise der WHO [10], des IASC [4] sowie des Internationalen Roten Kreuzes [5] zur Rolle von Führungskräften im Gesundheitssektor bei der Reduktion der psychischen Belastung ihrer Mitarbeiter*innen sind in Abb. 2 zusammengefasst, zusätzlich sind Aspekte aus einer früheren Publikation der WHO zum Schutz von Gesundheitsfachkräften bei Gesundheitsnotfällen berücksichtigt [8].

Dabei scheint zunächst wichtig, dass die Führungskräfte die psychische Belastung ihrer Mitarbeiter*innen neben den zahlreichen anstehenden Aufgaben im Blick behalten und eine Atmosphäre schaffen, in der den Mitarbeiter*innen das Gefühl vermittelt wird, sich mit Stress und psychischen Belastungen vertrauensvoll an ihre Vorgesetzten wenden zu können [4]. Dabei sollten Stress und psychische Belastungen ernst genommen und dem Schutz der Mitarbeiter vor diesen eine hohe Priorität eingeräumt werden [10]. Um Orientierung und Gefühle von Kontrolle und Selbstwirksamkeit zu fördern, sollten die Führungskräfte zudem darauf achten, ihren Mitarbeiter*innen alle relevanten Informationen zur Verfügung zu stellen und auf eine klare und eindeutige Kommunikation zu achten [4]. Zudem sollte sichergestellt werden, dass die Mitarbeiter*innen auch in Phasen hoher Arbeitsbelastung die Möglichkeit haben, Pausen und Erholungsmöglichkeiten wahrzunehmen und notwendige Maßnahmen der Selbstfürsorge durchzuführen [4]. Um die belastungsreduzierende Wirkung kollegialen Austausches nutzbar zu machen, sollten Führungskräfte darauf achten, ihren Mitarbeiter*innen Gelegenheiten zu verschaffen, sich mit anderen Teammitgliedern auszutauschen und so kollegiale Unterstützung zu fördern [2, 4]. Zudem sollten den Mitarbeiter*innen Zugangsmöglichkeiten zu psychosozialen und psychologischen Hilfsangeboten aufgezeigt werden, wobei insbesondere relevant ist, dass eine potenzielle Inanspruchnahme von Hilfsangeboten nicht mit einer Stigmatisierung verbunden wird [4]. Neben der Fürsorge für ihre Mitarbeiter*innen sollten Führungskräfte auch die eigene Selbstfürsorge im Blick behalten, da sie diesbezüglich wichtige Rollenvorbilder für ihre Mitarbeiter*innen darstellen [4]. Weiterhin sollten flexible Änderungen der Arbeitszeiten ermöglicht werden, falls Mitarbeiter*innen selbst oder enge Familienmitglieder von belastenden Ereignissen betroffen sind [10]. Eine wichtige Rolle kommt Führungskräften zudem bei der Vermittlung von Wertschätzung zu, da diese eine wichtige protektive Rolle bezüglich der psychischen Gesundheit in Krisenzeiten spielen kann [5]. Um Desorientierung und Hilflosigkeitsgefühlen vorzubeugen, spielt zudem die Etablierung klarer Rollen- und Aufgabenverteilungen eine wichtige Rolle, wobei diese regelmäßig an die aktuelle Lage angepasst werden sollten [8].

## Schlussfolgerungen

Gesundheitsfachkräften kommt im Rahmen der aktuellen Covid-19-Pandemie eine besondere Rolle bei der Bewältigung dieser Krise zu. Dabei treten zahlreiche unspezifische und spezifische Stressoren auf, die zu psychischen Belastungen bei den Gesundheitsfachkräften führen können. Um erste Handlungsanweisungen in der aktuellen Situation zur Verfügung zu stellen, wurden Empfehlungen internationaler Organisationen zusammengefasst. Dabei spielen für die Gesundheitsfachkräfte selbst insbesondere die Akzeptanz heftiger Emotionen, die Aufrechterhaltung von Gesundheitsverhalten und erfolgreichen Bewältigungsstrategien sowie sozialen Kontakten, die Berücksichtigung der Grundbedürfnisse sowie Unterstützung im Team oder durch Professionelle eine Rolle. Führungskräfte im Gesundheitssystem können eine wichtige Rolle bei der Reduktion psychischer Belastungen ihrer Mitarbeiter*innen spielen. Dabei spielt insbesondere das Vermitteln von Wertschätzung, Ernstnehmen psychischer Belastungen und Schaffen einer vertrauensvollen Atmosphäre, in der diese angesprochen werden können, die Förderung von Selbstfürsorge, kollegialem Austausch und professionellen Unterstützungsangeboten, das Zurverfügungstehen als Rollenmodell für Selbstfürsorge und die Etablierung einer klaren Kommunikation und klarer Verantwortlichkeiten eine Rolle. Die hier aufgeführten Hinweise stellen lediglich einen ersten Orientierungsrahmen bei der Bewältigung der aktuellen Covid-19-Pandemie dar und sollten durch die Etablierung umfassenderer Maßnahmen zur Förderung der körperlichen und psychischen Gesundheit von Gesundheitsfachkräften ergänzt werden.

## Fazit für die Praxis

- Gesundheitsfachkräfte sind im Rahmen der Covid-19-Pandemie mit zahlreichen Belastungen und Stressoren konfrontiert.
- Die Prävention und Reduktion psychischer Belastung von Gesundheitsfachkräften ist von zentraler individueller Bedeutung, spielt aber auch für die Aufrechterhaltung der Funktionsfähigkeit des Gesundheitssystems eine wichtige Rolle.
- Im Artikel werden erste Hinweise gegeben, was Gesundheitsfachkräfte und deren Führungskräfte tun können, um psychische Belastungen zu reduzieren.
- Dabei spielen eine Normalisierung von Stress und heftigen Emotionen, soziale Unterstützung, die Aufrechterhaltung von Grundbedürfnissen und Selbstfürsorge eine zentrale Rolle.
